# *NFATC2::NUTM2A/B* Fusions Characterize a Novel Indolent Myoepithelial‐Like Neoplasm of the Lungs and Salivary Glands

**DOI:** 10.1002/gcc.70083

**Published:** 2025-09-13

**Authors:** Abbas Agaimy, Josephine K. Dermawan, Elisabete Rios, Norbert Meidenbauer, Arno Dimmler, Robert Stoehr, Cristina R. Antonescu

**Affiliations:** ^1^ Institute of Pathology, Erlangen University Hospital Friedrich Alexander University of Erlangen‐Nuremberg Erlangen Germany; ^2^ Comprehensive Cancer Center European Metropolitan Area Erlangen‐Nuremberg (CCC ER‐EMN) Erlangen Germany; ^3^ Department of Pathology and Laboratory Medicine Memorial Sloan Kettering Cancer Center New York New York USA; ^4^ Department of Pathology and Laboratory Medicine Cleveland Clinic Cleveland Ohio USA; ^5^ Department of Pathology ULS São João Porto Portugal; ^6^ Department of Internal Medicine 5‐Hematology and Oncology, Erlangen University Hospital Friedrich Alexander University of Erlangen‐Nuremberg Erlangen Germany; ^7^ Institut und Praxis für Pathologie ViDia Christliche Kliniken Karlsruhe Germany

**Keywords:** lung, molecular profiling, myoepithelial carcinoma, NGS, nosology, sarcoma

## Abstract

With the increasing use of next‐generation sequencing, the classification of heretofore unclassified neoplasms is evolving rapidly. Specifically, gene fusions have emerged as context‐specific defining genetic markers for an increasing number of entities, mostly of soft tissue, bone, and salivary gland origin. We describe four myoepithelial‐like neoplasms of salivary (two) and pulmonary (two) origin, carrying recurrent *NFATC2* fusions involving *NUTM2B* (three) and *NUTM2A* (one) as fusion partners. Patients were two females and two males aged 24–67 years (median, 33). The tumor size ranged from 1 to 4.5 cm. Treatment was surgery without (three) or with (one) adjuvant radiochemotherapy. No metastases or other primary tumors were found at the time of diagnosis. Three patients with follow‐up (two with salivary, one with pulmonary tumor) were disease‐free at 9, 11, and 31 months. Original diagnoses were “unclassified neoplasm” with consideration of adamantinoma‐like Ewing sarcoma and myoepithelial neoplasm. Histology revealed infiltrating monotonous epithelioid to basaloid cells arranged into lobular aggregates, nests, and cords within variably sclerosed stroma containing extensive basement membrane‐like hyaline material. Frankly malignant features (malignant cytology, high mitotic activity, necrosis, perineural or lymphovascular invasion) were absent. IHC showed coexpression of low and high molecular weight keratins (AE1/AE3 and CK5/6; 4/4), EMA (2/2), and CD99 (2/2). Negative markers included p63 (0/4), NUT (0/4), S100 (0/4), SOX10 (0/4), p40 (0/2), and SMA (0/2). This study introduces a novel salivary and lung tumor entity driven by *NFATC2::NUTM2A/B* fusions and displaying myoepithelial‐like morphology but imperfect myoepithelial immunophenotype. Report of more cases should shed light on the biological properties and appropriate therapeutic strategies of this novel neoplasm.

## 1 Introduction

*NFATC2* and *NUTM2A/B* have been recognized recently as independent fusion partners in a variety of entities. Specifically, *NUTM2A/B* represent frequent fusion partners in subsets of high‐grade endometrial stromal sarcoma (ESS) [1], clear cell sarcoma of the kidney [2], CIC‐rearranged sarcomas [3], and emerging primitive undifferentiated pediatric sarcomas [4, 5]. With few exceptions [6], all these rare sarcomas display undifferentiated high‐grade morphology associated with an aggressive clinical course. *YWHAE* represents the most frequent fusion partner across these anatomically diverse entities, followed by CIC and *ATXN1* and *ATXN1L* [1, 2, 3, 4, 5, 6].

On the other hand, *NFATC2* is a definitional non‐ETS fusion partner of *EWSR1/FUS* genes in phenotypically and biologically highly heterogeneous lesions/neoplasms that include simple bone cysts [7, 8], subsets of epithelioid vascular tumors of bone [9], and a heterogeneous group of unclassified epithelioid and round cell neoplasms/sarcomas of bone and soft tissue [10, 11, 12, 13, 14]. To our knowledge, however, fusions involving *NFATC2* and *NUTM2A/B* have not been reported before as a distinct fusion in any defined entity. We herein describe detailed clinicopathological and molecular features of four neoplasms that are not classifiable into any of the established soft tissue, pulmonary, or salivary gland tumor entities, all carrying a novel *NFATC2* fusion.

## 2 Materials and Methods

Cases were identified in the consultation files of the authors. The tissue specimens were fixed in formalin and processed routinely for histopathology. Due to the consultation nature of most of the cases, immunohistochemistry (IHC) was performed in different laboratories, and the stains varied from case to case, based on tissue availability and initial differential diagnostic considetaions (details of the staining protocols and antibody sources are available upon request).

### 2.1 Next‐Generation Sequencing

RNA was isolated from formalin‐fixed paraffin embedded (FFPE) tissue sections using the RNeasy FFPE Kit of Qiagen (Hilden, Germany) and quantified spectrophotometrically using NanoDrop‐1000 (Waltham, United States). Molecular analysis was performed using the TruSight RNA Fusion panel (Illumina Inc., San Diego, CA, USA) with 500 ng RNA as input according to the manufacturer's protocol. Libraries were sequenced on a MiSeq (Illumina Inc., San Diego, CA, USA) with > 3 million reads per case, and sequences were analyzed using the RNA‐Seq Alignment workflow, version 2.0.1 (Illumina Inc., San Diego, CA, USA). The Integrative Genomics Viewer (IGV), version 2.2.13 (Broad Institute) was used for data visualization.

We declare that no Artificial Intelligence Generated Content (AIGC) tools such as ChatGPT and others based on large language models (LLMs) have been used in developing any portion of this manuscript.

## 3 Results

### 3.1 Clinical Features

The major findings were summarized in Table 1. Affected were two female and two male patients, aged 24–67 years (median, 33). Treatment was surgery without (3) or with (1) adjuvant radiochemotherapy. No metastases or other primary tumors were found at the time of diagnosis. The salivary tumor patients were disease‐free at 11 and 31 months. Case 4 was disease‐free at 9 months, while Case 3 was lost to follow‐up.

**TABLE 1:** Clinicopathologic and molecular findings in *NFATC2::NUTM2A/B* gene fusions salivary gland and pulmonary tumors.

No	Age/sex	Site/size (cm)	Treatment	Outcome	Fusion details
1	24/F	Lower lip minor salivary glands/1 cm	Surgery	NED (31 months)	*NFATC2 ex6::NUTM2B ex5*
2	33/F	Inner parotid lobe/4.5	Surgery + Ewing protocol CT + proton RT	NED (11 months)	*NFATC2 ex7:: NUTM2A ex4*
3	33/M	Lung/1.2	Surgery	NA	*NFATC2 ex6::NUTM2B ex5*
4	67/M	Lung UL/2.2	Surgery	NED (9 months)	*NFATC2 ex8::NUTM2B ex5*

Abbreviations: CT = chemotherapy, NA = not available, NED = no evidence of disease, RT = radiotherapy, UL = upper lobe.

### 3.2 Pathological Findings

**FIGURE 1:**
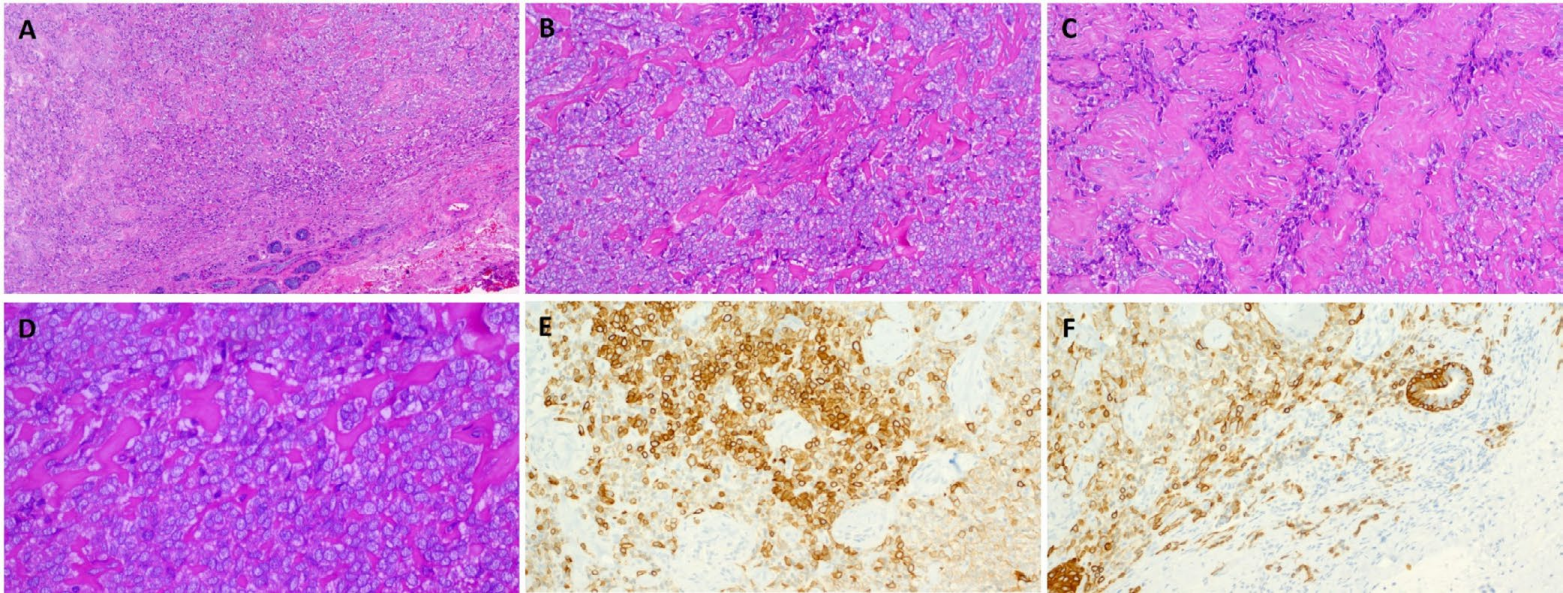
Representative example of *NFATC2::NUTM2B* fusion salivary gland neoplasms (Case 1). The neoplasm is well delineated from surrounding salivary lobules (A) and shows monomorphic basaloid epithelioid cells with a trabecular and nested pattern and variable stromal hyalinization (B, C). (D) Higher magnification shows rounded monomorphic bland cells in the more cellular areas. Expression of keratin AE1/AE3 (E) and CK5/6 (F), note focal infiltrative growth and entrapment of native salivary ducts in F.

**FIGURE 2:**
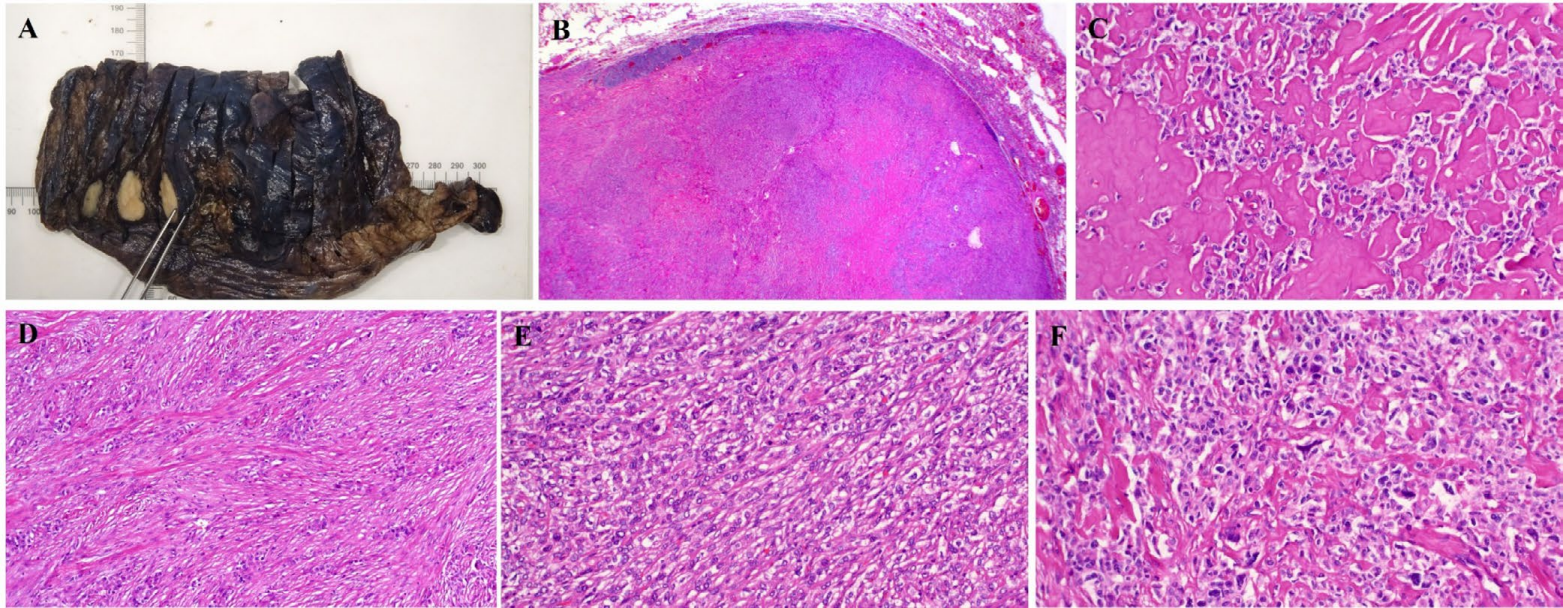
Representative example of *NFATC2::NUTM2B* fusion pulmonary neoplasms (Case 4). (A) Gross specimen showed well‐demarcated nodule with homogeneous tan‐yellow cut‐surface. (B) Low‐power view confirming well‐circumscribed but unencapsulated neoplasm with focal peripheral lymphoid cuffs and already at this low‐power recognizable prominent stromal hyaline deposits. (C) High‐power shows extensive basal membrane‐type hyaline deposits entrapping monomorphic pale‐stained epithelioid tumor cells. (D) Focal desmoplastic‐type stroma entrapping paucicellular tumor tissue are seen in both lung cases. (E) Highly cellular areas with little stroma and more ovoid to spindled cells. (F) A few scattered pleomorphic hyperchromatic cells were noted in this case.

The tumor size ranged from 1 to 4.5 cm (Tables 1 and 2). The original diagnoses were *unclassified neoplasm* with consideration of adamantinoma‐like Ewing sarcoma and myoepithelial neoplasm. Histology revealed infiltrating monotonous epithelioid to basaloid cells arranged into lobular aggregates, nests, and communicating cords within variably sclerosed stroma containing variable basement membrane‐like hyaline deposits. Mitotic activity was < 5 mitoses in 10 HPFs. No necrosis or perineural invasion was noted. IHC showed coexpression of low and high molecular weight keratins (AE1/AE3, CK5/6; 4/4), EMA (2/2), and CD99 (2/2). Negative markers include p63 (0/4), NUTM1 (0/4), S100 (0/4), SOX10 (0/4), p40 (0/2), and SMA (0/2) as well as many other tested markers based on the initial differential diagnostic consideration (Table 2). Representative examples of the salivary and pulmonary tumors are illustrated in Figures 1 and 2, respectively.

**TABLE 2:** Original diagnoses and immunohistochemical findings in *NFATC2::NUTM2A/B* fusion salivary gland and lung tumors.

No.	Original diagnosis	Pos IHC	Neg IHC
1	Unclassified basaloid (ALES?)	AE1/3, CD99, CK5/6, focal MUC4	CK7, S100, SOX10, p63, ALK D5F3, mammaglobin, pan‐TRK, NUT
2	Unclassified myoepithelial‐like	AE1/AE3, CK5/6, EMA, CD99	SMA, p63, CD34, CD31, CK7, ER, TRPS1, INSM1, GATA3, desmin, chromogranin, ER, S100, SOX10, p40, Pax8, NUT, CD56, synaptophysin, ALK, ERG, MUC4, pan‐TRK, CD30, SS18, STAT6, CDK4
3	Sclerosing myoepithelial‐like, Low‐grade	AE1/AE3, CK5/6, focal synaptophysin, CD56 and CD117	CK7, CK20, CK19, desmin, myogenin, ALK, NUT, p63, Pan‐Trk, SOX10, CD30, PAX8, SALL4, Oct3/4 und AFP. SMARCB1 and SMARCA4 retained
4	Unclassified, suggestive of *NFATC2::NUTM2A/B* fusion	AE1/AE3, EMA, CK5, EMA, focal CK18	Synaptophysin, SMA, desmin, STAT6, p63, D2‐40, chromogranin, S100, TTF1, p40, vimentin, CD99, SOX10, CK14, CK7, CD34, ERG, HMB45, ALK, MUC4, NUT, STAT6, TFE3.

Abbreviation: ALES = adamantinoma like Ewing sarcoma.

### 3.3 Molecular Findings

**FIGURE 3:**
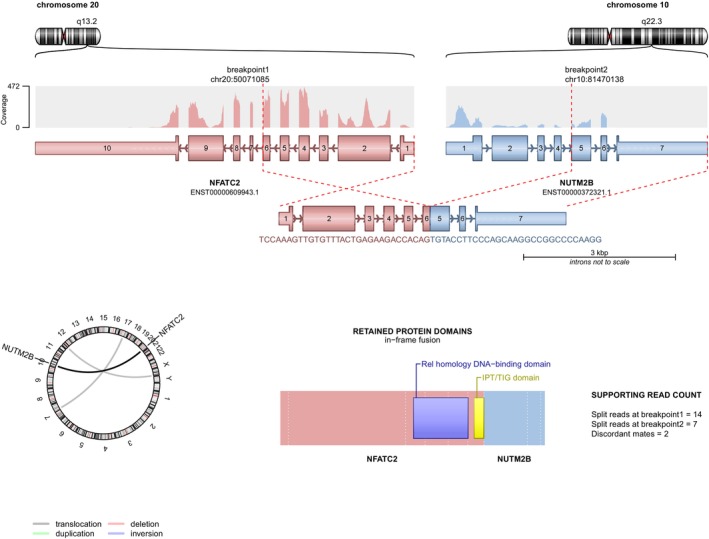
Schematic representation of gene fusions detected by panel sequencing. Illustrated is the gene structure of each fusion partner with the read coverage by panel sequencing, as well as the preserved exons resulting from the fusion event. The encoded chimeric protein including the retained protein domains is shown below. Depicted are gene fusions involving the genes *NFATC2* and *NUTM2B* (Case 3).

An in‐frame *NFATC2* fusion was detected in all four cases. The 3′ fusion partner was *NUTM2B* in three cases and *NUTM2A* in one. The breakpoints mapped to exon 6 (*n* = 2) and one each exons 7 and 8 of *NFATC2* fused to exon 5 of *NUTM2B* (*n* = 3; Figure 3) and exon 4 of *NUTM2A* (*n* = 1).

## 4 Discussion

Despite the wide implementation of different next‐generation sequencing tools in surgical pathology and in research studies, neoplasms carrying *NFATC2::NUTM2A/B* fusions have not been reported before, underlining the rarity of these fusions in general. To our knowledge, only a single case of a salivary gland neoplasm carrying an *NFATC2::NUTM2B* fusion has been included in a recent molecular survey study on unclassified head and neck neoplasms, but it was not clear if that case represents a novel entity or an incidental single observation [15].

This small series introduces a histologically distinct novel salivary gland and lung tumor entity driven by *NFATC2::NUTM2A/B* fusions and displaying variable myoepithelial‐like morphology but an imperfect myoepithelial immunophenotype. These *NFATC2::NUTM2A/B* fusion tumors exhibited histologic features that significantly overlap with those of myoepithelial neoplasms of salivary glands and soft tissue including typical cytology (epithelioid and ovoid to plump spindle cells) and myoepithelial‐like architectural patterns (solid sheets, lobules, variably sized nests, and branching/interconnected trabeculae within a background of sclerosed or desmoplastic stroma that contains variable amount of amorphic basement membrane‐like hyaline material). However, other features of myoepithelial neoplasms including plasmacytoid and frankly spindle cell morphology and myxoid stromal changes were absent. Moreover, ductal differentiation, abrupt squamous pearls/keratinized foci, or overt epithelial features were lacking.

Although all tumors showed some degree of infiltrative growth at the periphery, no frankly malignant features such as severe cytological atypia or pleomorphism, brisk mitotic activity, necrosis, lymphovascular invasion, or perineural invasion were present. At the immunohistochemical level, all four tumors expressed both broad spectrum and high molecular weight keratins (AE1/AE3, CK5/6), but lack squamous (p63/p40) and myoepithelial (S100, SOX10, SMA) markers. The monoclonal NUT antibody was negative in all cases.

The NUT member 2 (NUTM2) is a family of several gene paralogs that includes *NUTM2A* (10q23.2), *NUTM2B* (10q22.3), *NUTM2D* (10q23.2), *NUTM2E* (10q22.3), *NUTM2F* (9q22.32), and *NUTM2G* (9q22.33). Among these, *NUTM2B* followed by *NUTM2A* represents the most frequently involved members in oncogene fusions, while *NUTM2G* has been only recently encountered in subsets of NUT sarcomas defined by MYC‐associated factor X dimerization (MAD) protein family (*MXI1*, *MXD4*, and *MGA*) gene fusions, mostly fused to *NUTM1*, but rarely to *NUTM2A* and *NUTM2G* (*MXD4::NUTM2G* and *MXI1::NUTM2A* in one case each) [16]. Multifocal weak immunoexpression of NUTM1, probably representing antigenic cross‐reactivity with the NUTM2A protein, was observed in five cases [16].

The tumors we are describing herein differ from all prior *NUTM1/NUTM2A/B* fusion entities. They display similar histology in favor of a unique entity. Indeed, one lung case (Case 4; encountered during preparation of this study) was spotted initially based on morphological similarity to the other tumors. The bland morphology of these tumors, the lack of aggressive clinical features, and the uneventful although limited follow‐up in two cases are in line with a probably indolent clinical behavior. The one recently reported case (4.2 cm *NFATC2::NUTM2B* fused submandibular gland tumor in a 68‐year‐old male, reported as carcinoma with myoepithelial features and treated by surgery + adjuvant radiotherapy) had an uneventful course at 20 months follow‐up [15]. These observations are in line with the emerging notion that the probable cell of origin/histogenesis and exact fusion partners/breakpoints largely are reflected in the distinct clinical and biological properties of different neoplasms and that no single gene is by itself lethal [17].

Another controversial issue regarding these tumors is their histogenetic origin and their anatomic distribution. The myoepithelial‐like morphology of these tumors, their stromal characteristics, and their bland morphology are reminiscent of features seen in some *NFATC2::EWSR1/FUS* fusion neoplasms of bone and soft tissue [14]. This observation argues for *NFATC2* (and not the *NUTM2A/B* genes) being the major player in their oncogenesis/morphogenesis, but this remains speculative at the current time. Notably, we did not detect any immunoexpression of NUTM1 using the monoclonal antibody in contrast to the reported weak multifocal cross‐reactivity in *MAD::NUTM2* fusion sarcomas [16]. On another line, the lack of expression of S100, SOX10, and p63, particularly in the salivary cases, is a distinct feature compared to conventional salivary myoepithelial neoplasms. For this reason, we prefer reporting these tumors as “myoepithelial‐like” so that their separation from conventional myoepithelioma and myoepithelial carcinoma can help their delineation in the future. Moreover, the biologically non‐committed terminology “neoplasm” is preferred over calling them carcinomas or sarcomas to avoid the risk of overtreatment. Finally, whether this entity is limited to the lung and the salivary glands remains an issue for future studies as more cases are reported.

In summary, we presented four cases (two pulmonary and two salivary) of a distinctive low‐grade neoplasm characterized by bland histology, myoepithelial‐like morphology but with an imperfect myoepithelial immunophenotype, recurrent *NFATC2::NUTM2A/B* fusions, and likely an indolent clinical behavior. All findings are supportive of a novel entity. Future reports are needed to fully delineate the phenotypic and anatomic spectrum and the biological potential of this tumor type.

## Ethics Statement

Samples were used in accordance with ethical guidelines for the use of retrospective tissue samples provided by the local ethics committee of the Friedrich‐Alexander University Erlangen‐Nuremberg (ethics committee statements 24.01.2005 and 18.01.2012).

## Conflicts of Interest

The authors declare no conflicts of interest.

## Data Availability

The data that support the findings of this study are available on request from the corresponding author. The data are not publicly available due to privacy or ethical restrictions.
